# The Utility of the Supine-to-Stand Test as a Measure of Functional Motor Competence in Children Aged 5–9 Years

**DOI:** 10.3390/sports5030067

**Published:** 2017-09-12

**Authors:** Michael J. Duncan, Chelsey Lawson, Leanne Jaye Walker, David Stodden, Emma L. J. Eyre

**Affiliations:** 1 Faculty of Health and Life Sciences, Coventry University, Coventry CV1 5FB, UK; ac2444@coventry.ac.uk (C.L.); walkerl9@uni.coventry.ac.uk (L.J.W.); ab2223@coventry.ac.uk (E.L.J.E.); 2 Yvonne & Schuyler Moore Child Development Research Center, University of South Carolina, Columbia, SC 29208, USA; stodden@mailbox.sc.edu

**Keywords:** motor development, physical literacy, skill, test of gross motor development, functional movement

## Abstract

This study examined how supine-to-stand (STS) performance is related to process and product assessment of motor competence (MC) in children. Ninety-one children aged 5–9 years were assessed for process and product MC (10 m running speed and standing long jump) as well as process and product measures of STS. Tertiles were created for STS process and STS product scores to create 3 groups reflecting low, medium, and high STS competency. ANCOVA analysis, controlling for age, for process STS, indicated that process MC was significantly higher in children, classified as medium STS (*p* = 0.048) and high STS (*p* = 0.011) competence, and that 10 m run speed was slower for low STS compared to medium (*p* = 0.019) and high STS (*p* = 0.004). For product STS tertiles, process MC was significantly higher for children in the lowest (fastest) STS tertile compared to those in the medium highest (slowest) tertile (*p* = 0.01).

## 1. Introduction

The ability to rise from the floor to a standing position is considered a fundamental human motor skill [1] and is important for maintaining independence and mobility through adulthood [1,2]. As a consequence, the assessment of supine to standing is becoming more popular as a screening tool related to functional performance [2,3]. The supine-to-stand (STS) test is conceptualised as a combined assessment of flexibility [4], strength [5], locomotion and balance [2], and overall motor competence (MC) [3]. Due to its potential importance as a measure for assessing physical function and in informing strength and conditioning and rehabilitation programs across the lifespan [1,6], an examination of how STS relates to other aspects of MC and fitness is a needed step in establishing the utility of this test as a measure of functional MC.

The ability to manipulate one’s centre of mass and extremities to rise from the ground is employed in a variety of functional and recreational activities, including sports and exercise [7]. Subsequent research has demonstrated age-related differences in the performance of the STS task with the most advanced movement pattern being symmetrical (e.g., where both sides of the body move together in the same pattern) [8] most often occurring in older adolescents and young adults [9,10,11].

As people develop higher levels of physical function, their movement patterns also change accordingly. The process of these pattern changes has been captured and scored using component developmental sequences, [1] which have been used as one measure of STS performance and reflects the process of STS. Other authors have demonstrated that the time it takes to rise from the ground is linked to more advanced movement patterns [2,3], using STS time as a product measure of performance.

Most recently, Nesbitt, et al. [3] assessed component developmental sequences of STS in a sample of 122 3–5 year old children. Time to stand decreased as children got older. Nesbitt et al. [3] and Klima et al. [2] suggested that future research on product (i.e., time taken) and process (i.e., developmental sequences) aspects of STS was required to examine its utility as a holistic assessment of MC and its importance as a measure to predict functional capability.

The STS test offers potential as a global measure of functional MC, and emerging evidence suggests it is a useful and time-efficient tool that has clinical and practical relevance for lifespan movement assessment [2,3,6]. Competence in moving from a supine position to standing may also be a precursor to the development of other movement skills, yet only a limited number of studies have examined this measure in children, and none to date have examined both process and product measures alongside measures of motor competence. Understanding how STS performance might relate to other motor competence and function is needed as a necessary first step before any comprehensive guidance can be given regarding the predictive utility of STS. This study sought to address this issue.

## 2. Materials and Methods

### 2.1. Participants

Following institutional ethics approval, informed parental consent and child assent were obtained on 91 children (47 males, 44 females) aged 5–9 years (mean age ± SD = 6.8 ± 1.2 years) from two schools in central England that took part in this study. The schools were broadly representative of the schools within their geographical location. The school was within mid-range of electoral wards for deprivation and socio-economic status within the city. None of the children had any disabilities or special educational needs related to impaired motor development

### 2.2. Procedures

Data collection took place on two occasions separated by 48 h. On the first occasion, children’s anthropometrics and STS performance were assessed. On the second occasion, the remaining MC tests were assessed. Both process and product measurements of motor competence skills were examined in the present study to provide a holistic overview of MC. A process-oriented movement skill assessment are concerned with how the skill is performed (such as technique and sequencing of limb movement when running), whereas product-oriented movement assessment is concerned with the outcome or product of skill executions (such as distance jumped, thrown, time, and number of successful attempts) [12].

### 2.3. Anthropometry

Height (m) and body mass (kg) were assessed using a Seca Stadiometre and weighing scales (SECA Instruments, Hamburg, Germany). Body mass index (BMI) was then calculated as kg/m^2^.

### 2.4. Process Motor Competence Assessment

Four motor skills (two locomotor, two object control) were assessed using the Test of Gross Motor Development-2 (TGMD-2) scoring criteria [13]. In the current study, the following skills were assessed: run, jump, catch, and throw; these were collectively considered to provide an overall indication of locomotor and object control MC. These skills were selected as they are identified as the key motor skills required for development in school Physical Education in the United Kingdom [14]. Each skill comprises 3–5 components, with raters determining whether each component of each skill was present or not present, to determine the skill level of the child. Each skill was video-recorded (Sony video camera, Sony, Weybridge, UK) and subsequently edited into single film clips of individual skills on a computer using Quintic Biomechanics analysis software v21 (Quintic Consultancy Ltd., Sutton Coldfield, UK). The skills were then analyzed using the process-oriented checklist for each skill. Scores from two trials were summed to obtain a raw score for each skill. The scores for all the skills were then summed to create a process MC (scored 0–30) score. Scores from the run and jump were summed to create a locomotor competence score and the catch and throw summed to create an object control score following recommended guidelines of administration of the TGMD-2 [13]. Two researchers experienced in the assessment of children’s movement skills analyzed the TGMD-2 videos. Both raters were previously trained in two separate, 2–3 h sessions by watching videoed skills of children’s skill performances and rating these against a previously rated ‘gold standard’ rating. Intraclass correlation coefficients for inter and intra-rater reliability were 0.925 and 0.987, respectively.

### 2.5. Product Motor Competence Assessment

Two product measures—10 m sprint time and standing long jump—were assessed. Ten-metre sprint speed was assessed using smart speed gates (Fusion Sport, Coopers Plains, Australia). Two laser gates were set up 10 m apart, with the participant having a flying start to ensure that sprint speed (Secs) was measured independently of the acceleration phase. The fastest of three trials was used for data analysis. Standing long jump was assessed using a tape measure with the longest of 3 trials used for data analysis. Both assessments demonstrated acceptable test–retest reliability (ICC = 0.81–0.94).

### 2.6. Supine to Stand Performance

Supine-to-stand performance was assessed using procedures previously documented by Green and Williams [1] and Ng et al. [6]. The participants were asked to assume a supine position on a padded mat on the floor. They were asked to stand up as quickly as possible following a ‘go’ command from one of the research staff. To prevent influencing participants’ movement patterns, no demonstration was given [1]. Each participant was given a practice trial before undertaking two trials for data collection purposes [6]. Trials were video-recorded in the sagittal plane (Sony video camera, Sony, Weybridge, UK) for use in later analysis. On completion of data collection, the videos were uploaded to Quintic Biomechanics analysis software v21 (Quintic Consultancy Ltd., Sutton Coldfield, UK) for scoring. Two metrics of scoring supine-to-stand performance were employed. Firstly, the time taken to complete the supine to stand movement was taken as a product measure of this motor performance. The fastest time recorded from the two trials was used for subsequent analysis. Timing began at the onset of movement following the ‘go’ command and ceased when in stable stance without compensatory movement or sway and with both feet on the mat [1,6]. The supine to stand trials were also coded using VanSant’s [9,10] hypothesized component developmental sequences for the upper extremity (UE), axial region (AX) and lower extremity (LE). VanSant’s [9,10] movement components are scored out of 4 for each of the UE and AX and out of 5 for LE. Given that the process movement classification for STS performance offers numerical grading, where higher scores for each region of the body reflect more optimal/competent movement, scores for each region were summed across both trials. In this way, we sought to compile a measure of STS performance, congruent with the philosophy for scoring of the TGMD-2. This resulted in possible scores of 6–26, with six representing the least developed movement patterns and 26 the most advanced movement patterns. On completion, tertiles were created for STS process scores and STS time (product) scores in order to create 3 groups reflecting low, medium, and high STS process competence and low, medium, and high STS product competence. Intraclass correlation coefficients for inter- and intra-rater reliability were 0.90 and 0.95 for the coding of STS developmental sequences, respectively, and 0.92 and 0.98 for STS time assessment, respectively.

### 2.7. Statistical Analysis

Pearson’s product moment correlations, controlling for age, were employed to examine relations between BMI, STS process and product measures, and MC process and product measures. In order to examine any differences in process MC scores, standing long jump distance and 10 m running speed, a 2 (gender) by 3 (high, medium, and low STS competence) way analysis of covariance (ANCOVA), controlling for chronological age and BMI was used. Thus, 6 separate ANCOVAs (3 for STS process scores and 3 for STS time) were conducted, with process MC, standing long jump distance, and 10 m running speed as the dependent variables. In this way, any differences between the dependent variables as a result of gender, STS process scores, or STS time, or their interaction, could be determined whilst also accounting for any association between the dependent variable and the covariates [15]. Where any significant differences were detected, Bonferroni post-hoc comparisons were used to indicate where those differences lay. The truncated product method [16] was used to combine all the *p*-values in this study to determine whether there was a bias from multiple hypothesis testing. The truncated product method *p*-value was <0.0001, indicating that the results are not biased by multiple comparisons. Statistical significance was set at *p* = 0.05, and all analysis was completed using the Statistical Package for Social Sciences (SPSS) version 24.

## 3. Results

Results from Pearson’s product moment correlations, controlling for age, indicated significant relations (all *p* < 0.001) between all variables with the exception of TGMD2 scores and BMI (*p* > 0.05). The age adjusted correlation matrix is presented in Table 1.

### 3.1. The Supine to Stand Process

When scores for the STS developmental components were used as the dependent variable (reflecting STS process competence), results from ANCOVA analysis indicated that, for process MC, there were no significant higher-order interactions, nor was BMI significant as a covariate (both *p* > 0.05). Age was however significant as a covariate (*p* = 0.015, partial η^2^ = 0.08, β = 1.076), indicating that every 1 year increase in age was associated with just over a 1 point increase in process MC score. 

**Figure 1:**
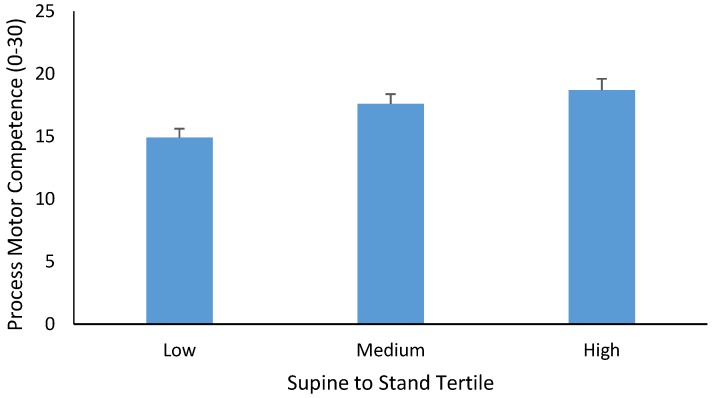
Mean ± SE of the process MC score (0–30) in children categorized as low, medium, and high for STS developmental sequences.

Gender was also significant as a main effect (*p* = 0.005, partial η^2^ = 0.104) with boys having higher process MC scores compared to girls. The mean ± SE of the process MC score was 18.3 ± 0.59 and 15.8 ± 0.62 for boys and girls, respectively. There was also a significant main effect for STS tertiles (*p* = 0.009, partial η^2^ = 0.125, see Figure 1). Bonferroni post-hoc multiple comparisons indicated that the process MC score was significantly lower for children in the lowest STS tertile compared to children in the medium STS tertile (*p* = 0.048) and children in the high STS tertile (*p* = 0.011). There were, however, no significant differences in the process MC score between children in the medium and high STS tertile (*p* = 0.989).

When data were analyzed using product measures of MC, ANCOVA analysis for standing long jump indicated no significant higher-order interactions, nor was BMI significant as a covariate (both *p* > 0.05). There were also no significant differences in standing long jump distance according to STS tertile (*p* = 0.305). Age was significant as a covariate (*p* = 0.001, partial η^2^ = 0.138, β = 9.309), indicating that every 1 year increase in age was associated with just over a 9 cm increase in standing long jump distance. Gender was also significant as a main effect (*p* = 0.004, partial η^2^ = 0.101) with boys having a longer standing long jump compared to girls. Mean ± SE was 125.1 ± 3.7 for boys and 109.2 ± 3.8 for girls. 

**Figure 2:**
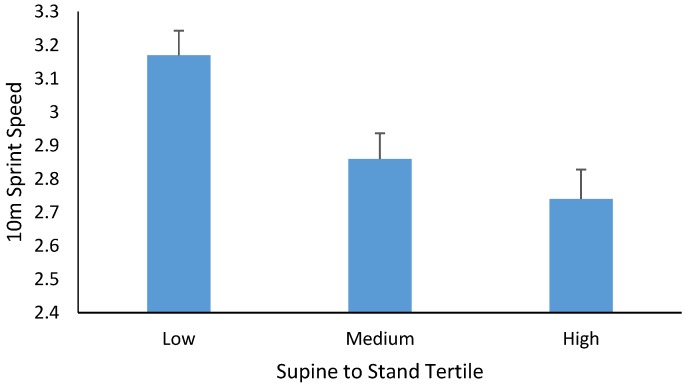
Mean ± SE of 10 m sprint speed (s) in children categorized as low, medium, and high for STS developmental sequences.

For the 10 m running speed, there was also no significant higher-order interaction (*p* > 0.05). Age was significant as a covariate (*p* = 0.001, partial η^2^ = 0.213, β= −0.189), as was BMI (*p* = 0.04, partial η ^=2^ = 0.056, β = 0.031), where increasing age was associated with a faster 10 m sprint time and an increasing BMI was associated with a slower 10 m sprint time. Boys had significantly (*p* = 0.003, partial η^2^ = 0.119) faster 10 m sprint times than girls (2.79 ± 0.05 s for boys vs. 3.06 ± 0.06 s for girls), and there was also a significant main effect for STS tertiles (*p* = 0.003, partial η^2^ = 0.153, see Figure 2). Bonferroni post-hoc multiple comparisons indicated a significantly lower 10 m speed for those in the lowest STS tertile compared to those in the mid (mean diff = 0.309, *p* = 0.019) and high (mean diff = 0.419, *p* = 0.004) tertiles. There was no significant difference in 10 m run speed between those in the mid and high STS tertiles (mean diff = 0.115, *p* = 0.889).

### 3.2. The Supine-to-Stand Product

**Figure 3:**
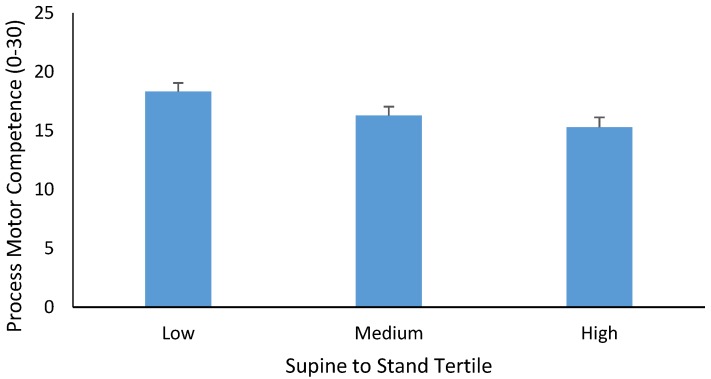
Mean ± SE of process MC (0–30) in children categorized as low (faster), medium, and high (slower) for STS time.

When STS time (product) tertiles were considered in the analysis with the process MC score as the dependent variable, findings were similar to those reported for STS process scores. There was no higher-order interaction, nor was BMI significant as a covariate (both *p* > 0.05). Age was significant as a covariate (*p* = 0.001, partial η^2^ = 0.276, β = 2.105). There was also a significant main effect for STS time tertiles (*p* = 0.04, partial η^2^ = 0.087, see Figure 3). Post-hoc analysis indicated that process MC was significantly higher for children in the lowest (fastest) STS tertile compared to those in the mid-highest (slowest) tertile (*p* = 0.01). There was no difference in process MC between those in the middle and highest tertiles (*p* = 0.719) or the lowest and middle tertiles for STS time (*p* = 0.210).

When the product measures or MC, standing long jump and 10 m sprint speed were used as dependent variables, the findings remained similar for STS product (time) compared to STS process. ANCOVA analysis for standing long jump indicated no significant higher-order interactions, nor was BMI significant as a covariate (both *p* > 0.05). There was also no significant difference in standing long jump distance according to STS time tertile (*p* = 0.335). Age was significant as a covariate (*p* = 0.001, partial η^2^ = 0.294, β = 13.5), indicating that every 1 year increase in age was associated with a 13.5 cm increase in standing long jump distance. For the 10 m running speed, there was also no significant higher-order interaction, nor was there a significant main effect for STS tertiles (both *p* > 0.05). Age was significant as a covariate (*p* = 0.0001, partial η^2^ = .439, β = −0.285), as was BMI (*p* = 0.01, partial η^2^ = 0.088, β = 0.041), where increasing age was associated with a faster 10 m sprint time, and increasing BMI was associated with a slower 10 m sprint time. As was described in the section on STS process tertiles, a 10 m sprint time remained significantly different between gender groups (*p* = 0.002, partial η^2^ = 0.122) with boys being faster than girls.

## 4. Discussion

This study examined the association between STS performance using both a process and product approach and MC using process (TGMD-2) and product (10 m running speed and standing long jump) measures. This study is one of the first to examine the utility of STS performance as a measure of functional MC in children and the first to examine its association with measures of MC related to sport participation and performance. Children who scored higher on process measures of STS performance also scored higher on process measures of MC and were significantly faster in terms of 10 m sprint time as a measure of product MC. These findings add support to assertions previously made [3] that the STS test is a measure of functional MC in children. 

The results presented here are novel in that this is the first study to compare both process and product measures of STS performance with both process and product measures of MC in a pediatric population. The time taken to rise from STS demonstrates concurrent validity with salient measures of MC used in the assessment of children’s MC with findings from the current study agreeing with prior work on the topic [2,6].

The original validation of the STS test in adults [9,10,11] described developmental movement sequences that individuals performed to move from supine to standing. Green and Williams [1] then presented a numerical classification of these patterns segmented into different regions (upper and lower extremity and axial regions). Other studies have solely recorded the time taken to rise from supine to standing, suggesting that it is a global measure of functional MC [2,6]. In the current study, the developmental sequence scores for STS were summed, using the same process as established measures of MC [13], to provide one overall measure of the process of STS performance. This is also akin to the philosophy of popular composite measures of functional movement, such as the functional movement screen [17]. When used in this way, the results of the present study evidenced significant associations with both process and product measures of general motor competence, evidencing concurrent validity of STS performance as a measure of motor competence. Overall, using concurrent process- and product-oriented assessments of the same skills has been identified as key in comprehensively capturing the level of MC in human movement [6,18]. Data presented by Ng et al. [6] also suggested that a combination of process and product measures for STS performance was needed to properly use this movement as a test of global MC, despite only reporting on STS time in their study. Data from this study support that STS could be practically applied as a screening tool for functional MC, fulfilling the suggestion of Logan et al. [18] for a measure of both process and product measures in settings such as school physical education and youth strength and conditioning in a time- and labor-efficient manner. The results of the present study align well and support prior work using product and process STS performance in children [3] and product only measures of STS performance [6]. Despite this, due to the lack of other work that has presented data on the STS test, it is difficult to draw comparisons in children. Given the data presented in the current study and those presented by others [3,6], an important next step is to integrate STS assessment in other work examining children’s motor development and its association with other health- and performance-related variables.

This study is, however, not without its limitations, including the cross-sectional design and constraints on causal interpretations. The study was based on a convenience sample of children and further scrutiny of associations between the STS test and other skills and fitness measures is needed before conclusive statements can be made regarding its use of a measure of global functional MC. In the current study, process MC was based on scores form run, jump, catch, and throw as taken from the TGMD-2 [13], and product MC scores were based on results of the 10 m sprint speed and the standing long jump. These measures were used as they represent key motor skills required for a majority of athletic tasks and for physical education in school [14], and the objective of the present study was to explore whether STS performance was associated with these key motor skills. We acknowledge that there is a need to examine how STS performance might be associated with other measures of MC (e.g., hopping, galloping, kicking, and bouncing a ball) and other measures of gross motor skills (e.g., fundamental motor skills) and fitness such as muscular strength, agility, and power. It would also be useful to examine how STS performance, both from a process and product perspective, change as a consequence of training intervention.

## 5. Conclusions

The results of this study suggest that the STS test can be used as a measure of functional MC in children aged 5–9 years. Both the time taken to rise from supine to standing (product) and the movement pattern (developmental sequences) of rising from supine to stand differentiate MC in children.

## Figures and Tables

**Table 1:** Pearson product moment correlation matrix (adjusted for age) showing the relationship between product and process measures of MC, product and process measures of supine-to-stand (STS) performance, and BMI. ** *p* < 0.01

Measure Taken	BMI (kg/m^2^)	Standing Long Jump Distance (cm)	10 m Running Speed (s)	Supine to Stand Process (out of 28)	Supine to Stand Time (s)
Process Motor Competence (0–30)	−0.180	0.628 **	−0.634 **	0.457 **	−0.463 **
BMI (kg/m^2^)		−0.308 **	0.411 **	−0.408 **	−0.508 **
Standing Long Jump Distance (cm)			−0.713 **	0.369 **	−0.414 **
10 m Running Speed (s)				−0.532 **	0.539 **
STS Process (out of 28)					−0.525 **
